# Infant brain activity in response to yawning using functional near-infrared spectroscopy

**DOI:** 10.1038/s41598-019-47129-0

**Published:** 2019-07-23

**Authors:** Shuma Tsurumi, So Kanazawa, Masami K. Yamaguchi

**Affiliations:** 1 Department of psychology, Chuo University, 742-1, Higashinakano, Hachioji, Tokyo 192-0393 Japan; 2 Japan Society for the Promotion of Science, Chiyoda-ku, Tokyo 102-0083 Japan; 3 Department of psychology, Japan Women’s University, 1-1-1, Nishi-ikuta, Tama-ku, Kawasaki, Kanagawa 214-8565 Japan

**Keywords:** Perception, Human behaviour

## Abstract

Yawning is contagious in human adults. While infants do not show contagious yawning, it remains unclear whether infants perceive yawning in the same manner as other facial expressions of emotion. We addressed this problem using functional near-infrared spectroscopy (fNIRS) and behavioural experiments. We confirmed behaviourally that infants could discriminate between yawning and unfamiliar mouth movements. Furthermore, we found that the hemodynamic response of infants to a yawning movement was greater than that to mouth movement, similarly to the observations in adult fMRI study. These results suggest that the neural mechanisms underlying yawning movement perception have developed in advance of the development of contagious yawning.

## Introduction

Many studies have shown that contagious yawning can be observed in several mammals^1–10^. Provine^7^ showed that 16 of 30 human adults yawned as a response to videos of a person yawning, while only 7 of 30 adults yawned as a response to videos of a person smiling. Contagious yawning in humans begins to emerge at around age four years^10^. Massen *et al*.^3^ showed that, in chimpanzees, the proportion of contagious yawning in response to yawning videos was significantly higher than that of yawning in response to videos showing chimpanzees’ daily behavior. Joly-Mascheroni *et al*.^2^ showed that dogs yawned in response to human yawning more than to non-yawning mouth movement. These results from humans and nonhuman suggest that contagious yawning is a strong phenomenon regardless of the species among mammals.

On the other hand, it has been reported that contagious yawning seemed not to be observed in human infants. Millen and Anderson^9^ investigated contagious yawning in infants, and reported null results; they found that only 3 of 22 infants yawned in response to videos showing their mothers yawning. This result suggests that human infants are not generally susceptible to contagious yawning. Considering the developmental trajectory of yawning behavior, spontaneous yawning is observed even in fetuses^11^. Subsequently, first year infants might discriminate yawning, and then until the preschool age, the contagious yawning behavior would continue to develop according to the development of the primary motor cortex.

Although no prior studies succeeded to find contagious yawning in infancy, there are a lot of studies showing significant sensitivity to various facial expressions in infancy^12,13^. For instance, LaBarbera *et al*.^12^ found that 4-month-old infants could discriminate between joyful and neutral faces, and Kotsoni *et al*.^13^ showed that 7-month-olds have categorical perception of facial expressions of emotion. To our knowledge, however, the discrimination of yawning faces during infancy has not yet been explored. The present study examined whether infants could discriminate yawning from mouth movement, and showed higher activation of the areas around the superior temporal sulcus (STS) in response to the presentation of yawning.

Using fNIRS is one of the most effective methods for investigating the neural correlates of face processing in young infants. A recent series of fNIRS studies measuring the hemodynamic response in infants reported that the temporal areas of the brain were involved in face processing. Otsuka *et al*.^14^ showed that the concentration of oxyhemoglobin (oxy-Hb) in the right temporal area of 5- to 8-month-old infants was higher during the presentation of upright faces than during the presentation of inverted faces. In addition, Nakato *et al*.^15^ investigated view-invariant face processing in infancy using fNIRS, and found that 8-month-old infants showed greater neural activity in the posterior temporal areas in response to frontal and profile faces. These neural activations were related to the discrimination of faces. Furthermore, it was found that the infants’ posterior temporal areas are activated when perceiving facial expressions of emotion and biological motion. Ichikawa *et al*.^16^ presented upright and inverted dynamic point-light displays (PLDs) depicting facial expressions of surprise to 7- to 8-month-old infants; higher activation in the right temporal area was observed only during the upright presentation of the dynamic PLDs. Nakato *et al*.^17^ reported a higher activation in infants’ right posterior temporal area in response to the static angry faces, and in the left area to the static happy faces. Based on the above evidence, we hypothesized that the posterior temporal areas would be activated when viewing the yawning movement.

An adult functional magnetic resonance imaging (fMRI) study showed that the bilateral STS was sensitive to yawning movement. Schürmann *et al*.^8^ found a significantly higher blood oxygen level- dependent (BOLD) signal in the right posterior STS and bilateral anterior STS while viewing yawning movement compared to that in response to other mouth movements. Thus, we expected that we would be able to identify specific brain activity in response to the presentation of yawning faces during infancy.

In this study at first, we conducted behavioral experiments using the preferential looking paradigm to examine the infants’ discrimination of yawning. We also tested the inversion effect of the yawning movement in infants as a part of the behavioral experiment. Next, we measured the activity in the bilateral temporal areas of 5- to 8-month-old infants during the presentation of yawning and mouth movements. We hypothesized that yawning movement induces higher activity in the bilateral temporal areas than other mouth movements.

## Experiment 1a

The aim of this experiment was to investigate whether infants could discriminate between yawning and mouth movements. If yawning movement is a face specific movement for infants, infants would be expected to show the preference for yawning similar to that for facial expressions of emotion as the index of discrimination of yawning and other facial movements.

### Results

**Figure 1:**
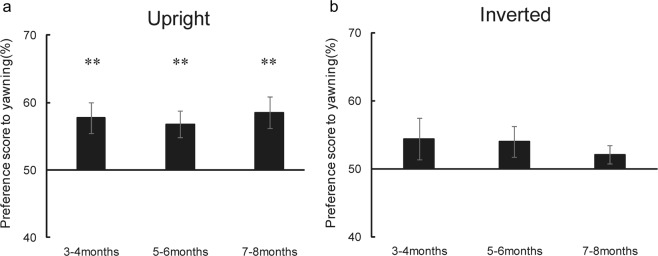
Results of Experiment 1. (**a**) Preference scores for the yawning movements in upright condition (Experiment 1a). (**b**) Preference scores for the yawning movements in inverted condition (Experiment 1b). Error bars represent ± 1 SEM. **p < 0.01 (vs.chance).

We calculated an individual preference score for the yawning movements. The preference scores were calculated by dividing the infant’s looking time for the yawning movement across the 4 trials by the total looking time across the 4 trials and then multiplying this ratio by 100 (Fig. 1a). To test whether infants prefer yawning movement, we conducted a two-tailed *t*-test on the preference score (vs. chance level of 50%). This analysis revealed that 3- and 4-month-olds, 5- and 6-month-olds, and 7- and 8-month-olds significantly preferred yawning movement to mouth movement (*t*(14) = 3.38, *p* = 0.004, *d* = 0.872; *t*(14) = 3.43, *p* = 0.004, *d* = 0.885; and *t*(14) = 3.65, *p* = 0.002, *d* = 0.943, respectively). We also conducted a repeated-measure analysis of variance (ANOVA) on the percentage of time spent looking at each of the stimuli, by setting age (3–4, 5–6, and 7–8 months) as between-participant factor and movement condition (yawning and mouth movements) as within-participant factor. The ANOVA revealed a significant main effect of movement condition (*F*(1,42) = 36.35, *p* < 0.00, *ηp*^2^ = 0.46), suggesting that all aged infants preferred yawning over mouth movement. No significant main effect of age and interaction was observed (*F*(2,42) = 0.00, *p* = 0.1, *ηp*^2^ = 0.00; *F*(2,42) = 0.15, *p* = 0.86, *ηp*^2^ = 0.01); there is no difference between ages.

This result indicated that infants of all age groups could discriminate yawning from mouth movement. However, there is one alternative possibility that infants simply preferred the particular motion pattern of the yawning stimuli rather than the actual facial expression of yawning. To test this possibility, we investigated the infants’ preference when yawning and mouth movement were presented upside down. If the preference to yawning movement would result from the preference to the motion specific to faces, the preference would disappear when it was presented inverted. We tested this possibility in the next experiment.

## Experiment 1b

The aim of this experiment was to test whether the preference to yawning movement result from the perception of facial movement specific to yawning. Previous studies have shown that the preference to face-like stimuli disappeared when they were presented inverted^18^, and the preference to upright faces was significantly higher than that to inverted faces^19^. We predicted that if yawning movements are a special movement of the face, we found no preference to inverted stimuli.

### Results

We calculated an individual preference score for the yawning movements as the same manner used in Experiment 1a. Figure 1b shows the mean preference scores obtained in Experiment 1b. To test whether infants showed a significant preference to yawning movement, we conducted a two-tailed *t* test on the preference score in each age group. This analysis revealed no significant preference to yawning movements in 3- and 4-month-olds (*t*(14) = 1.46 *p* = 0.167, *d* = 0.376), 5- and 6-month-olds (*t*(14) = 1.75 *p* = 0.102, *d* = 0.452), and 7- and 8-month-olds infants (*t*(14) = 1.52, *p* = 0.149, *d* = 0.394). In addition, we conducted a repeated-measure ANOVA on the percentage of time spent looking at each of the stimuli, by setting age (3–4, 5–6, and 7–8 months) as between-participant factor and movement condition (yawning and mouth movement) as within-participant factor. No significant main effect of age and interaction was observed (movement condition: *F*(1,42) = 2.78, *p* = 0.10, *ηp*^2^ = 0.06; age: *F*(2,42) = 0.00, *p* = 0.1, *ηp*^2^ = 0.00; interaction: *F*(2,42) = 1.93, *p* = 0.16, *ηp*^2^ = 0.08); there is no difference between movement condition and age, suggesting that the preference for yawning and mouth movement did not differ in an inverted face.

Furthermore, we conducted a two-way analysis of variance (ANOVA) with two factors, namely orientation of stimuli (upright or inverted) and age (3- and 4-months, 5- and 6-months, or 7- and 8-months), on the preference score of the yawning movements in order to confirm whether the preference to yawning movements in the upright presentation was higher than in the inverted presentation. A significant effect of orientation of stimuli was observed (*F*(1,84) = 5.07, *p* = 0.026, *ηp*^2^ = 0.057), There were no main effects of age (*p* = 0.936) nor of any interaction (*p* = 0.688).

One may still argue that the preference for yawning movement in Experiment 1a would be modulated by the greater attention given to an upright than an inverted face. To exclude this possibility, we tested it by comparing the total looking time between upright face (Experiment 1a) and inverted face (Experiment 1b). A repeated-measure ANOVA, with two between-participant factors, face orientation (upright and inverted), and age (3–4 and 5–6, 7–8 months) revealed no significant main effect and interaction (face orientation: *F*(1,42) = 2.80, *p* = 0.10, *ηp*^2^ = 0.06; age: *F*(2,42) = 1.85, *p* = 0.17, *ηp*^2^ = 0.08; interaction: *F*(2,42) = 2.42, *p* = 0.10, *ηp*^2^ = 0.10); there is no difference in attentional modulation between an upright and inverted face. This result suggests that the preference for yawning movement is not modulated by the greater attention afforded to an upright face.

These results showed that the preference to yawning movement disappeared when it was presented inverted. Taken together, our findings suggest that infants can discriminate between yawning movement and mouth movement.

## Experiment2

### Results

Data from 12 infants, from whom more than 3 valid trials each for the yawning movement and mouth movement conditions could be obtained, were used for the analysis. The mean number of trials was 5.08 for the yawning movement condition and 5.33 for the mouth movement condition.

**Figure 2:**
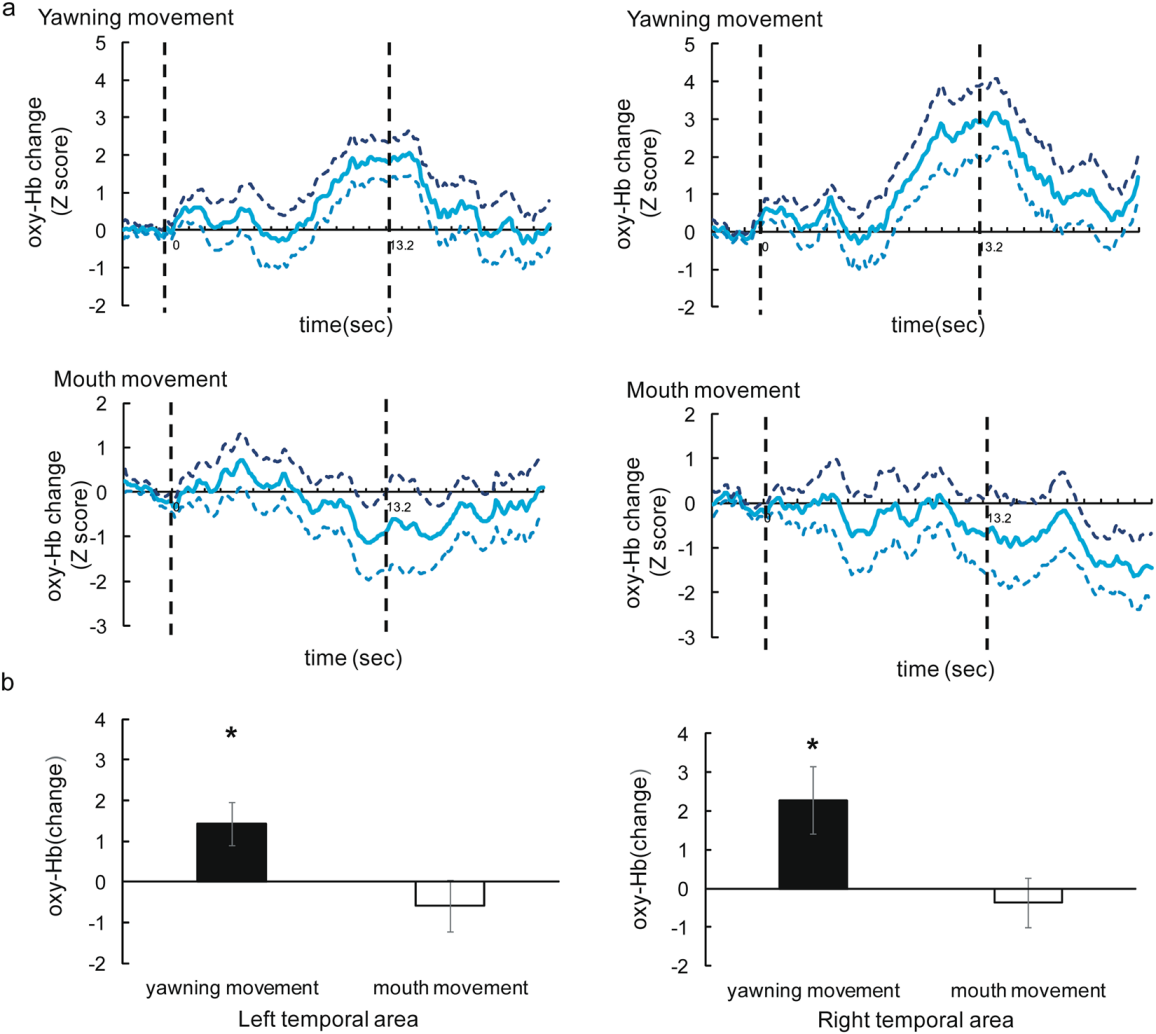
Results of the NIRS measurement in infants. (**a**) The time course of the average change in oxyhemoglobin (oxy-Hb) in 5- and 8- month- olds during the yawning movement and mouth movement conditions. The left column shows the hemodynamic changes in left temporal area, and the right column shows the hemodynamic changes in right temporal area. The thick line in graph represents the mean Z score, and broken line represent the range of ±1 standard of error of mean (SEM). On the horizontal axis, 0 represents the beginning of the presentation of both movements and 13.2 represents the end of the presentation of both movements; the vertical dashed line at 0 and 13.2 s denote the onset and offset of the test stimulus presentation, respectively. (**b**) Mean Z scores during the 8–14 s presentation in the left and right temporal areas. The error bars represent ±1 SEM. In yawning movement condition, the concentrations of oxy-Hb in both temporal areas were significantly greater than the chance level of 0. *p < 0.05.

Figure 2a shows the time course of the average change in the oxy-Hb concentration during the presentation of the yawning and mouth movements (results of deoxy Hb and total-Hb changes are provided in theSupplementary Fig. S1 and S2). The concentration of oxy-Hb in both left and right temporal areas increased during the presentation of yawning movement, but not during the presentation of mouth movement.

Figure 2b shows the mean Z score of oxy-Hb from the left and right temporal area during the period extending from 8–14 s. We conducted a two tailed one- sample *t*-test against a chance level of 0 (baseline) for each condition. The results revealed that the concentration of oxy-Hb and total-Hb increased significantly in both temporal areas during the presentation of yawning movement (for oxy-Hb: left, *t*(11) = 2.70, *p* = 0.020, *d* = 0.779 and right, *t*(11) = 2.65, *p* = 0.022, *d* = 0.766, for total-Hb: left, *t*(11) = 2.81, *p* = 0.017, *d* = 0.810 and right, *t*(11) = 3.05, *p* = 0.011, *d* = 0.880) No significant increase was observed in both temporal area during the presentation of mouth movement (for oxy-Hb: left, *t*(11) = −0.918,*p* = 0.378, *d* = 0.265; right, *t*(11) = −0.562, *p* = 0.585, *d* = 0.162; and for total-Hb: left, *t*(11) = 0.524,*p* = 0.610, *d* = 0.151; right, *t*(11) = 0.451, *p* = 0.660, *d* = 0.130). We found no significant increase of deoxy-Hb in temporal areas in either condition (*p* = 0.250).

Subsequently, to examine the difference in activation between yawning and mouth movement, we conducted a repeated measures analysis with the facial movement (yawning or mouth movement) and hemisphere (left or right) as within-subject factors on the average Z-score of oxy-Hb, deoxy-Hb, and total-Hb. We found a significant effect of facial movement on oxy- and total-Hb, but not deoxy-Hb (oxy-Hb: *F*(1,22) = 15.89, *p* < 0.01, *ηp*^2^ = 0.42; total-Hb: *F*(1,22) = 9.58, *p* < 0.01, *ηp*^2^ = 0.30; deoxy-Hb: *F*(1,22) = 0.01, *p* < 0.01, *ηp*^2^ = 0.00). No other significant effect or interaction were found for oxy-, deoxy-, and total-Hb (all *ps* > 0.10). Thus, yawning and mouth movement showed a significant difference.

**Figure 3:**
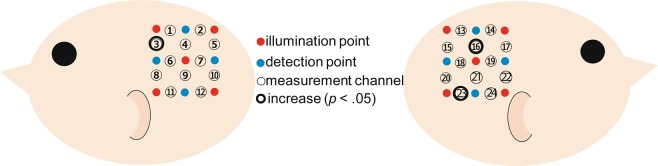
Locations of the measurement channels in the current study. The probes for infants were placed on the right and left temporal regions centered at the T5 and T6 position of the international 10–20 system. The blue circles represent for detector, and the red circles represent for emitter. Each number correspond to the measurement channels.

A further analysis was conducted to investigate the cortical area that exhibited the activation of yawning or mouth movement (Fig. 3). Then we conducted one sample t-test against the zero value (baseline) on the Z-scores of oxy-Hb for each channel separately. We found that the cortical activation of yawning movement in ch3, ch16, and ch23 were significantly higher than zero (*t*(11) = 2.85, *p* = 0.016, *d* = 0.821; *t*(11) = 3.16, *p* = 0.009, *d* = 0.913; *t*(11) = 3.28, *p* = 0.007, *d* = 0.947). On the other hand, we did not find significant cortical activation to mouth movement in any channels.

We finally conducted the analysis on the looking time to each yawning and mouth movement in valid trials in fNIRS experiments to exclude the concern that infants just observed upright yawning more than upright mouth movement. This result showed no difference of looking time between yawning and mouth movement (*t*(11) = 1.08, *p* = 0.30; average of looking time to yawning in one trial was 11617.71, and to mouth movement in one trial was 11241.41).

In contrast to previous fNIRS studies which found the main response to faces in the right temporal area^14–16^, we found a significant increase in the oxy-Hb concentration in both temporal areas during the presentation of yawning movement. These results suggest that both temporal areas are involved in the processing of yawning movement.

## Discussion

In the current study, we examined infants’ discrimination of yawning by using (a) the preferential looking paradigm and (b) fNIRS measurement. In Experiment 1, we explored the infants’ looking preference to yawning and mouth movement in 3- to 8-month-old infants. We found that infants of all age groups showed significant preference to yawning movement, but not to mouth movement in the upright condition (Experiment 1a). However, the preference to yawning movement disappeared when the faces were inverted (Experiment 1b). These results suggest that infants could discriminate between yawning and mouth movements. In Experiment 2, we measured the brain activity of 5- to 8-month-old infants in response to the presentation of yawning and mouth movements using fNIRS. Our results showed that the concentration of oxy-Hb increased significantly in the bilateral temporal areas during the presentation of yawning movement, compared with the presentation of objects (vegetables). However, no such brain activity was observed during the presentation of mouth movements.

The results of the fNIRS experiment revealed that the concentration of oxy-Hb significantly increased in bilateral temporal areas during the presentation of yawning movement. This finding is consistent with the adult fMRI study^8^ demonstrating greater bilateral activation in the bilateral superior temporal sulcus in response to yawning movement. In this study, we measured the hemodynamic changes in the bilateral temporal regions near the STS area. The previous studies suggest that these temporal areas are involved in face processing^14–16^. Based on these results, it is suggested that the bilateral temporal areas are involved in the processing of yawning movement even in infants.

There are two possible reasons behind the greater activation in bilateral temporal areas to the presentation of yawning in infants. First, this activation could be related to the discrimination of yawning movement, same as in adults^8^. Second, the familiarity of the stimuli might influence the neural activation in infants’ temporal areas. There are two types of familiarity; one is short-term, formed as a result of habituation, and the other is long-term, formed due to daily exposure. The former is formed by habituation during the experimental procedure. Previous fMRI studies also used this familiarity to the neural adaptation procedure, which attenuated the neural activation by the repeated presentation of identical stimuli^20^, and this procedure was recently used in infant fNIRS face studies^21^. The latter is formed by the daily exposure to specific faces such as the mother’s face and other adult female faces^22,23^. Recent fNIRS studies showed that the presentation of mother and adult female faces induced higher neural activation^22,23^. Our results of infants’ neural activity of yawning processing reflect this discrimination of yawning movement and the formed long-term familiarity.

The neural activity in the bilateral temporal areas of 5- to 8-month-old infants induced by viewing yawning movement indicates that the neural mechanism underlying the processing of yawning movements would develop at least by 5 months of age. This finding is consistent with the development of the ability of face processing^14,17,24^. The neural activity of temporal areas in response to upright faces was observed even in 5- to 8-months of age^14^, and the activity of the temporal area induced by facial expressions has been observed in 6- to 7-month-old infants^17^. Leppänen and Nelson^24^ also indicated that the neural mechanisms underlying the processing of facial expressions would develop between 5 and 7 months of age. These previous results and our results suggest that the developmental period of discrimination of yawning and facial expressions is overlapped.

The results of the behavioral experiments showed that infants could discriminate yawning from other mouth movements. Furthermore, the discrimination of yawning movement was impaired when the yawning movement was inverted, reflecting the face inversion effect^25–28^. These two results suggest that infants could discriminate yawning as a facial movement rather than as a low-level visual movement.

In the current study, we found that infants who did not demonstrate contagious yawning could discriminate yawning movement. Contagious yawning is related to the social brain regions such as the STS and the mirror-neuron systems^29^. A recent study showed that the primary motor cortex is the important region for contagious yawning rather than the mirror-neuron systems^30^. Considering the developmental trajectory of yawning, spontaneous yawning is observed even in fetuses^11^. After this spontaneous behavior, our study showed that infants aged below one year could discriminate yawning. Until the preschool age, the contagious yawning behavior would continue to develop according to the development of the primary motor cortex^10^. The neuro-developmental trajectory of contagious yawning in older toddlers and children is a matter for future study.

In our study, we investigated the perception of yawning in infants. As a result, we found that infants could discriminate yawning from mouth movement, and also showed the greater activation to yawning in infants’ bilateral temporal areas. These results suggest that the development of neural mechanisms of yawning perception precedes the development of contagious yawning.

## Methods

### Experiment1a

#### Participants

In total, 15 3- and 4-month-old (10 boys and 5 girls, mean age = 101.6 days, range from 80–121 days), 15 5- and 6-month-old (9 boys and 6 girls, mean age = 164.67 days, range from 135–194 days) and 15 7- and 8-month-old (6 boys and 9 girls, mean age = 235.6 days, range from 203–254 days) infants participated in the experiment. An additional 6 infants were tested but were excluded from the analysis due to a side bias of more than 90% (*n* = 4) or failure to look at the monitor for more than 60% (*n* = 2) of the duration of the experiment. The infants were recruited through newspaper advertisements. All infants were full-term at birth and healthy at the time of the experiment. Written informed consent was obtained from the parents of the participants.

#### Stimuli

We recorded videos of both the movements with the help of 8 actors (4 men and 4 women), and selected the videos made by 2 of the female actors as the experimental stimuli because they produced a more natural yawning movement compared to others. As a control movement, we used the non-nameable mouth movement gesture developed by Schurmann *et al*.^8^. In this latter mouth movement, the actors opened the mouth, and moved the tongue sideways against the cheek. The size of the stimuli was approximately 18.14° × 23.88° in visual angle. Both yawning and mouth movements consisted of 180 frames, each of which was presented for 33 ms successively; the total duration of each movement was about 6 seconds.

#### Apparatus

All the stimuli were displayed on a CRT monitor at a resolution of 1024 × 768 pixels controlled by a computer. The CRT monitor was placed inside an enclosure made of iron poles and covered with cloth. The infant sat on his or her parent’s lap in front of the CRT monitor, which was located approximately 40 cm away. There were two loudspeakers, one on either side of the CRT monitor. A charge-coupled device (CCD) camera was located just below the monitor screen. The infant’s behavior throughout the experiment was recorded digitally through this camera. The experimenter could observe the infant’s behavior via a monitor connected to the CCD camera. The infants watched the stimuli passively while their brain activity was measured, and they were allowed to watch the stimuli for as long as they were willing to.

#### Procedure

The preferential looking paradigm was used to measure each infant’s response. Before the experiment session started, the parent was instructed not to look at the CRT monitor during trials to prevent parent’s looking behavior from affecting the infant looking behavior. Each experimental session consisted of 4 12-s trials. At the beginning of each trial, a cartoon with a brief sound was presented at the center of the monitor to attract the infant’s attention. The experimenter initiated each trial as soon as the infant began paying attention to the cartoon. In each trial, a yawning movement and a mouth movement enacted by the same actor, were presented side by side twice (12 s) on the CRT monitor. The order of the presentation of two female actors and the position of yawning movement and mouth movement were counterbalanced across infants. The duration of the trials was fixed, regardless of whether or not the infant looked at the stimuli.

#### Data analysis

One observer, who was naive to the position of the stimuli, measured each infant’s looking time to the right and left field from the video recordings. We calculated a score on individual preference for yawning and mouth movement, by dividing the infant’s looking time for yawning or mouth movement across the four trials by the total looking time across the four trials and then multiplying this ratio by 100.

### Experiment1b

The Methods in Experiment 1b were the same as those in Experiment 1a except for the followings.

#### Participants

In total, 15 3- and 4-month-old (6 boys and 9 girls, mean age = 110.3 days, range from 80–131 days), 15 5- and 6-month-old (7 boys and 8 girls, mean age = 171.87 days, range from 135–193 days), and 15 7- and 8- month-old (5 boys and 10 girls, mean age = 235.8 days, range from 213–254 days) infants participated in the experiment. The infants were recruited through newspaper advertisements. All the infants were full-term at birth and healthy at the time of the experiment. Written informed consent was obtained from the parents of the participants.

#### Stimuli

The yawning and mouth movements used in Experiment 1a were inverted. The duration and the size of the stimuli were same as those used for Experiment 1a.

### Experiment2

The Methods in Experiment 2 were the same as those in Experiment 1a except for the followings.

#### Participants

The participants in this study were 12 healthy 5- and 8-month-olds (9 boys and 3 girls, mean age = 198.16 days, ranging from 161–234 days). We decided this sample size based on previous NIRS study^14–17,21–23^. All the infants were full-term at birth and healthy at the time of testing. An additional 4 infants were tested, but excluded from the final sample because of an insufficient number of successful trials for analysis (fewer than 3 trials for either the yawning movement or mouth movement condition) due to fussiness. The infants were recruited through newspaper advertisements. This study was approved by the ethical committee of Chuo University, and written informed consent was obtained from the parents of the infant. The experiments were conducted in accordance with the Declaration of Helsinki guidelines.

#### Stimuli and design

Movie showing yawning and mouth movements were presented during the test periods. The yawning movements and mouth movements were presented on alternating trials. The order of presentation was counterbalanced across infants. The duration of the test period was fixed at 13.2 s, and the duration of the baseline period was over 13.2 s.

The size of yawning movements and mouth movement videos was approximately18.14° × 23.88° of visual angle. Both the movement videos consisted of 90 frames, each of which was presented for 33 ms successively; the duration of both the movements was about 2970 ms. In each trial, we presented yawning or mouth movements 4 times, in which the movements of the two female actors were shown alternately. The order of two female movements was alternated across the test period. In order to draw and maintain the attention of the infants, a small red cross (fixation point) was presented 330 ms along with a beeping sound before both movements began. The size of the fixation point was approximately 9.55° × 9.55°.

**Figure 4:**
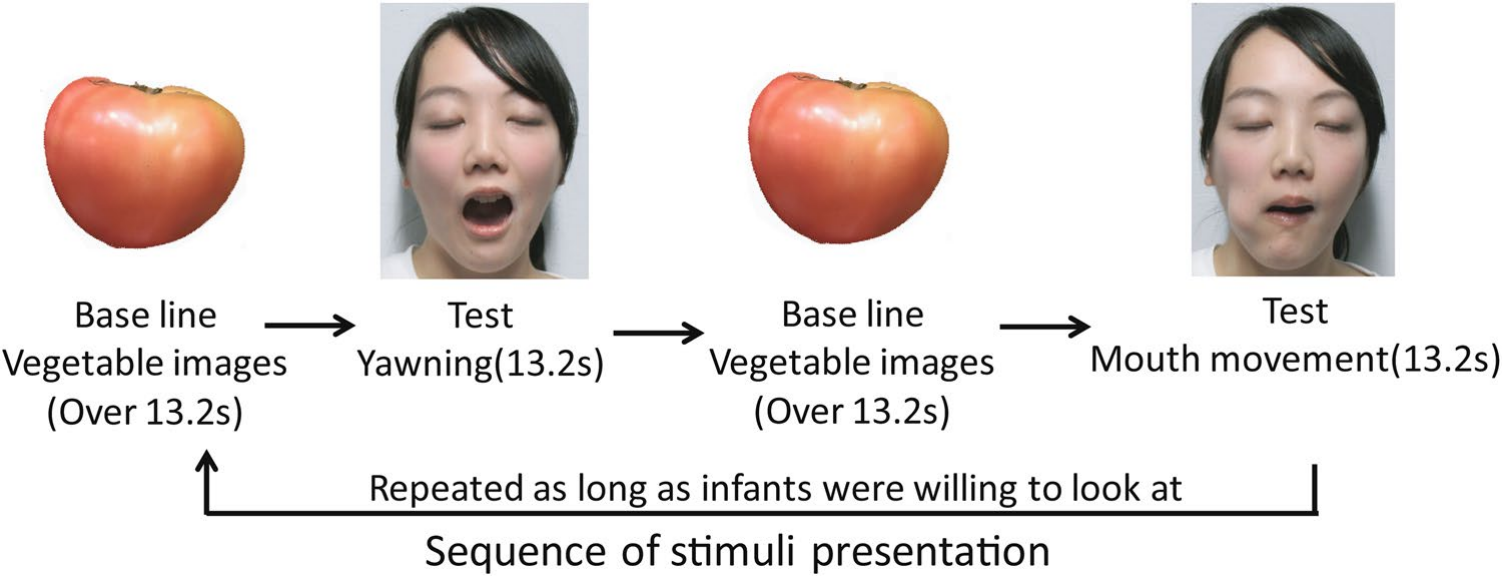
The timeline of the stimulus presentation. In each trial, images of five kinds of vegetables were presented during the baseline period for at least 13.2 s. In the test period, yawning or mouth movements were presented for 13.2 s. The presentation order of yawning and mouth movement in test period was counterbalanced across infants.

For the baseline period, five images of vegetables were shown in a random order. Each of these images was shown for about 990 ms following the presentation of a small red cross, which was the same as that used in the test period. The intertrial interval was controlled by the experimenter, and its duration was at least 13.2 s. The hemodynamic responses obtained during looking at the objects were used as a baseline (Fig. 4).

#### Recording

Brain activity was recorded using a Hitachi ETG-4000 device system (Hitachi Medical,Japan), which can record NIRS data from 24 channels simultaneously, with 12 channels for the right temporal area and 12 for the left. The instrument generated two wavelengths of near-infrared light (695 and 830 nm) and measured the time courses of the levels of oxy-Hb, deoxy-Hb, and total-Hb from the 24 channels at a temporal resolution of 0.1-s. We used fNIRS sensor probes which was made especially for infants (Hitachi Medical, infant probe 3 × 3 mode) for the recording sessions, and most of the infants appeared comfortable during experiments and were not reluctant to participate since these probes have a lower weight and make softer contact with the skin than previous probes. We used a pair of probes, each containing 9 optical fibers (3 × 3 array). Of the 9 fibers, 5 were used as emitters and 4 were used as detectors. The optical fibers of each probe were kept in place using a soft silicon holder. The distance between the emitters and the detectors was set at 2 cm. Each pair of adjacent emitting and detecting fibers formed a single measurement channel, and this allowed the measurement of oxy-Hb and deoxy-Hb changes in 12 channels for each hemisphere.

In each hemisphere, the position of the probes covered the temporal area centered at T5 and T6 according to the international 10–20 electrode system^31^. The posterior regions targeted in the current study are comparable to the regions found to be responsive to the yawning movement in a previous fMRI study with adults^8^. When the probes were positioned, the experimenter checked to see if the fibers were keeping in touch with the infant’s scalp appropriately. The Hitachi ETG-4000 system automatically detects the channel if the placement is inadequate to measure the emerging photons from each channel.

Compared with fMRI, it is difficult for the fNIRS method to identify the specific neural areas. However, we measured the bilateral temporal face-specific areas at positions T5 and T6, in which previous infant fNIRS studies observed greater neural activation to face stimuli^16^. Based on previous results, we believed we could measure the neural activity in bilateral temporal face-specific areas near the STS region.

#### Data analysis

We removed the trials from the analysis (1) if the infants did not look at the test stimuli (yawning movements and mouth movements) for over 8 s, or (2) if they became fussy. Moreover, (3) the trials in which the infants looked back at the face of the experimenter during the preceding baseline period, and (4) trials in which movement artifacts were detected by the analysis of sharp changes in the fNIRS raw time series were removed from the analysis.

The raw oxy-Hb, deoxy-Hb, and total-Hb data from individual channels were digitally filtered with 0.02- to 1-Hz bandpass filter to remove longitudinal signal drift and noise from the instrument. Next, the mean concentration recorded from each channel was calculated for each subject by averaging the data across the trials in a time series (at a temporal resolution of 0.1-s) starting 3 s before the trial onset up to 10 s after trial offset. We calculated Z scores for oxy-Hb, deoxy-Hb, and total-Hb concentration in the yawning movement and mouth movement conditions, respectively, for each channel for each subject on the basis of the mean concentrations in the time series. The Z scores (z) were calculated as the difference between the mean concentrations during the baseline (m_1_) and trial phases (m_2_), divided by the standard deviation of the baseline data (s):

$$z=({m}_{2}-{m}_{1})/s,$$

The mean concentration value recorded 3 s immediately before each test trial was used as a baseline concentration, similar to the protocol used in a previous study^16^. Although the NIRS raw data were originally relative values and could not be averaged directly across participants or channels, the normalized data such as the Z scores could be averaged, regardless of the unit of measurement^32–34^. The Z scores obtained from 12 channels within each measurement area were averaged.

We performed statistical analyses on the mean Z scores from 8 s to 14 s after a stimulus onset. Previous studies on infants have shown that a latency of up to 10 s in oxy-Hb was observed by presenting dynamic facial images (11 s–17 s: Ichikawa *et al*.^16^; 10 s–18 s: Lloyd-Fox al^35^). This finding suggests that the peak of neural response to dynamic stimuli was expected to emerge later after a stimulus onset. As expected, this period, namely, from 8 s to 14 s, included the range of maximum changes observed across infants for oxy-Hb. A two-tailed one-sample t-test on a chance level of 0 (baseline) was conducted for the mean Z scores in the left and right temporal areas during the period of 8 s–14 s.

## Supplementary information

Supplementary material
