# Supplementary material for: Infant brain activity in response to yawning using functional near-infrared spectroscopy
Source: Sci Rep. 2019 Jul 23;9:10631. doi: 10.1038/s41598-019-47129-0 (PMC6650597; doi:10.1038/s41598-019-47129-0)
Supplement: Supplementary file 1 — Supplementary material [file 41598_2019_47129_MOESM1_ESM.docx]

Infant brain activity in response to yawning using near-infrared spectroscopy

Shuma Tsurumi^1,2^ , So Kanazawa^3^ , Masami K. Yamaguchi^1^

1. Department of psychology, Chuo University, 742-1, Higashinakano, Hachioji, Tokyo 192-0393, Japan
2. Japan Society for the Promotion of Science, Chiyoda-ku, Tokyo 102-0083, Japan
3. Department of psychology, Japan Women’s University, 1-1-1, Nishi-ikuta, Tama-ku, Kawasaki, Kanagawa 214-8565

Corresponding concerning this article should be addressed to Shuma Tsurumi

E-mail: perry.super178@gmail.com

**Supplemental material**

**
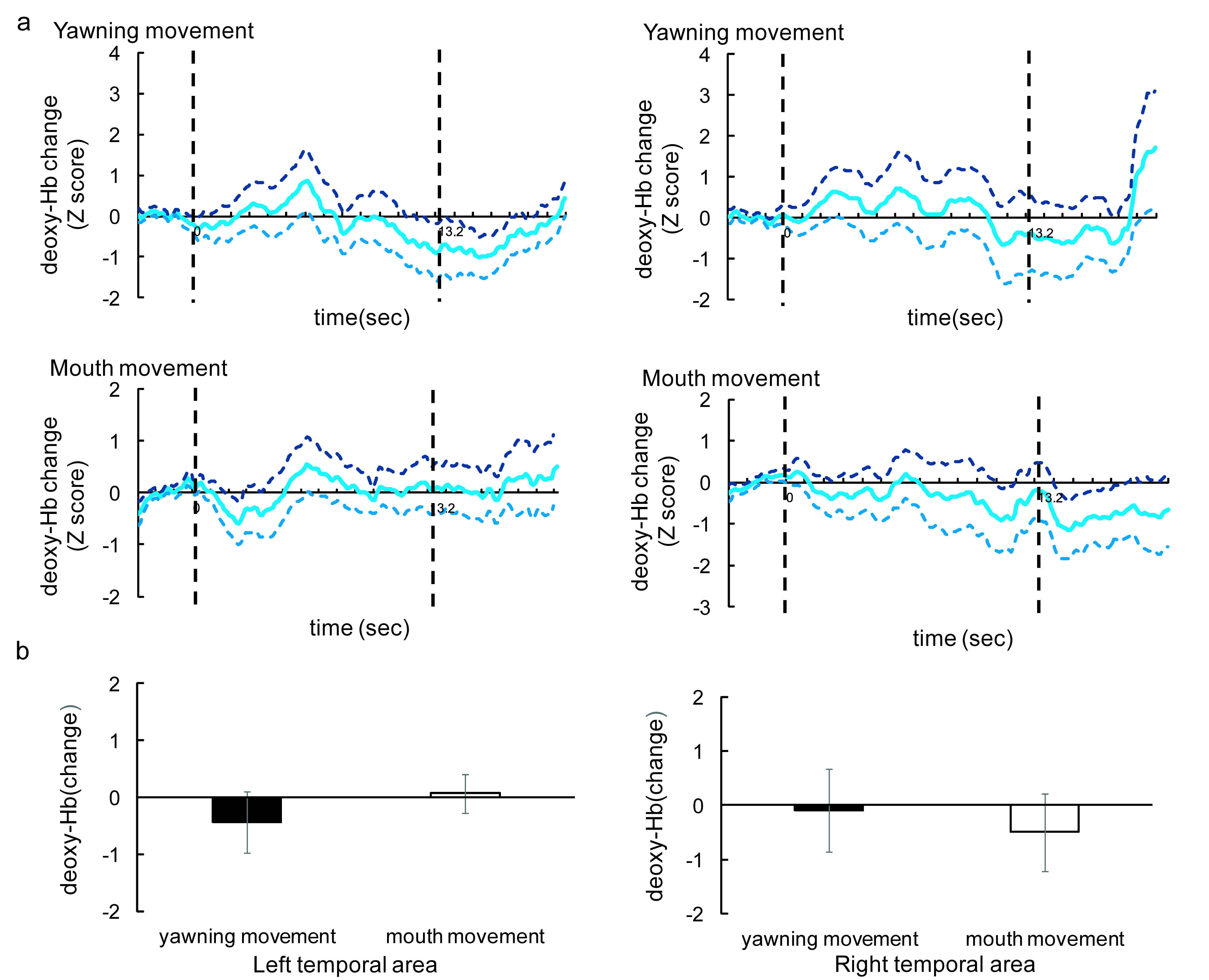
**

**Fig.S1.** Results of the NIRS measurement in infants. (a) The time course of the average change in deoxy-Hb in 5- and 8- month- olds during the yawning movement and mouth movement conditions. The left column shows the hemodynamic changes in left temporal area, and the right column shows the hemodynamic changes in right temporal area. The thick line in graph represents the mean Z score, and broken line represent the range of ±1 SEM. On the horizontal axis, 0 represents the beginning of the presentation of both movements and 13.2 represents the end of the presentation of both movements; the vertical dashed line at 0 and 13.2 s denote the onset and offset of the test stimulus presentation, respectively. (b) Mean Z scores during the 8-14s presentation in the left and right temporal areas. The error bars represent ±1 SEM. No significant increase was found in both movements.

**
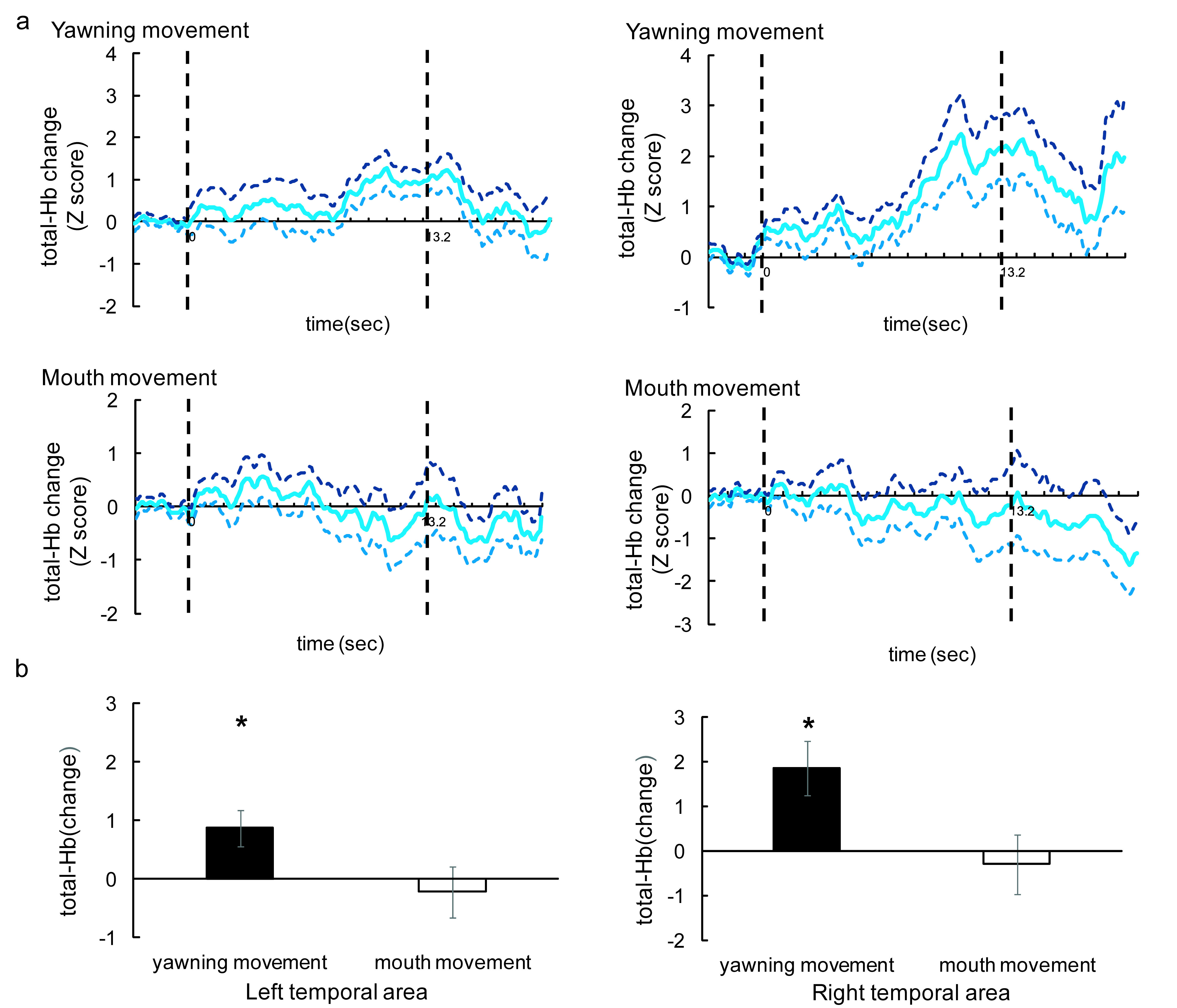
**

**Fig.S2.** Results of the NIRS measurement in infants. (a) The time course of the average change in total-Hb in 5- and 8- month- olds during the yawning movement and mouth movement conditions. The left column shows the hemodynamic changes in left temporal area, and the right column shows the hemodynamic changes in right temporal area. The thick line in graph represents the mean Z score, and broken line represent the range of ±1 SEM. On the horizontal axis, 0 represents the beginning of the presentation of both movements and 13.2 represents the end of the presentation of both movements; the vertical dashed line at 0 and 13.2 s denote the onset and offset of the test stimulus presentation, respectively. (b) Mean Z scores during the 8-14s presentation in the left and right temporal areas. The error bars represent ±1 SEM. In yawning movement condition, the concentrations of total-Hb in both temporal areas were significantly greater than the chance level of 0. * *p* < .05.
